# Future insights of pharmacological prevention for AKI post cardiopulmonary bypass surgery (based on PK/PD approach)

**DOI:** 10.3389/fphar.2022.975641

**Published:** 2022-09-22

**Authors:** Dias Permeisari

**Affiliations:** Department of Clinical Pharmacy, Airlangga University, Surabaya, Indonesia

**Keywords:** AKI post-CPB, pharmacological intervention, technological improvement, PK/PD approach, pk profile-related CPB

## Abstract

The incidence of acute kidney injury (AKI) post-cardiopulmonary bypass (CPB) can cause an increase in the rate of renal replacement therapy (RRT) and mortality rate. Compared to brain and liver damage post-CPB, AKI has the highest incidence of 83%. Based on this phenomenon, various efforts have been made to reduce the incidence of AKI post-CPB, both pharmacologically and non-pharmacologically interventions. The purpose of this review is to emphasize several renal protector agents which under optimal conditions can provide significant benefits in reducing the incidence of AKI post-CPB. This article was obtained by conducting a study on several kinds of literature, including the original article, RCT study, systematic review and meta-analysis, and other review articles. There are five renal protector agents that are the focus of this article, those are fenoldopam which effectively works to prevent the incidence of AKI post-CPB, while furosemide has shown satisfactory results in patients with decreased renal function when administered in the Renal Guard (RG) system, mannitol, and nitric oxide, both of these can also effectively reduce the incidence of AKI post‐CPB by controlling its blood concentration and timing of administration, and another form of N-Acetylcysteine, namely N‐Acetylcysteine amide has better activity as a renoprotective agent than N‐Acetylcysteine itself. The benefits of these agents can be obtained by developing devices that can control drug levels in the blood and create optimal conditions for drugs during the use of a CPB machine.

## Highlights

**•**  In addition to the problem of dosage, the selection of drugs that are in accordance with the patient’s condition and the time of administration is a crucial to get the benefits of a drug
**•**  The use of a CPB machine can affect the pharmacokinetic parameters of a drug, including drug levels in the blood, and this will also affect the effectiveness of the drug, thus requiring device development that can control drug levels in the blood and adjust the dose during CPB
**•**  Renoprotective agent can turn into a nephrotoxin if it exceeds the effective range of drug levels in the blood

## Introduction

Based on a clinical study that has been conducted, the incidence of acute kidney injury after cardiac surgery is the most common adverse outcome with an incidence rate of up to 83% by the AKIN and KDIGO criteria (da Silva et al., 2021), when it compared to the prevalence of brain injury was 20% and for acute liver injury in the first 48 h was 0.77% (Tachibana et al., 2021; Zakeri et al., 2021). Afterward, the definition of AKI which is used in this review is in accordance with the KDIGO criteria, characterized by an increase in serum creatinine (SCr) 0.3 mg/dl within 48 h and a decrease in urine output (UO) < 0.5 ml/kg every hour for 6 h. Generally, the incidence of AKI might occur after cardiac surgery as a consequence of CPB utilization. In a retrospective study involved 3889 patients, present a correlation between duration use of CPB and incidence of acute kidney injury after cardiac surgery (Axtell et al., 2020), it was part of the intraoperative factors that causes incidence of AKI post-CPB through several mechanisms of action, namely hemodynamic perturbation, mechanical factors, oxygen delivery, carbon dioxide production, and inflammation (de Somer et al., 2011). In addition to those intraoperative factors, there is another condition of preoperative factor that also possible to increase the incidence of AKI post-CPB which commonly caused by the presence of pre-existing kidney disease (Alramadan et al., 2019), pulmonary disease, obesity (Thakar et al., 2005; Fu et al., 2021) and older age (Thakar et al., 2005; Chronopoulos et al., 2010). Various strategies were made to prevent incidence of AKI related to CPB utilization. The preventive strategies for AKI post-CPB can be classified into two, those are the pharmacological and non-pharmacological intervention (Ostermann et al., 2021). Based on several studies that have been conducted, including randomized controlled trial (RCT), review article, and systematic review and meta-analysis, only a few numbers of these pharmacological interventions may provide promising results in reducing the risk of developing AKI post-CPB (Buylaert et al., 1989; Yang et al., 2014; Gillies et al., 2015; Hu et al., 2019; Krawczeski Catherine, 2019).

## Furosemide

Furosemide is a loop diuretic that works by decreasing the activity of the sodium-potassium-chloride cotransporter in the apical membrane of the tubular epithelial cells in the thick ascending loop of Henle so as to reduce the medullary net oxygen consumption (Khan et al., 2022). On the other hand, furosemide administration can also cause volume depletion and possibly renal hypoperfusion (Ho and Power, 2010). In anuric patients, furosemide administration becomes ineffective because in that condition glomerular filtration stops, so furosemide becomes completely inactive (Patschan et al., 2019). Based on a study conducted by Zhao *et al*, showed that furosemide can reduce in-hospital mortality and improve renal function recovery in patients with AKI urine output (UO) stage 2–3, however furosemide administration in patients with AKI SCr stage 2–3 and chronic kidney disease become ineffective (Zhao et al., 2020). One strategy to achieve optimal conditions for the mechanism of furosemide as a renoprotective agent by maintaining fluid balance is to carry out technological improvements of device such as the Renal Guard (RG) System (Barbanti et al., 2015; Luckraz et al., 2017; Katoh et al., 2019; Luckraz et al., 2021). The results in Table 1.

**TABLE 1:** Evidences of renal guard system utilization for AKI prevention.

Study, year	Type of surgery	Study design	Dosage regimens	Results
Luckraz et al. (2017)	Cardiac surgery	Prospective, observational	20 mg, 90 min preprocedure-during procedure-4 h after last contrast inj	No patients developed AKI within 36 h of surgery
Luckraz et al. (2021)	Cardiac surgery	RCT	20 mg initiated, cont.10 mg/h, Start after intubation until 6 h after CICU admission	Reduced AKI incidence, increases urine output within 24 h of surgery
Barbanti et al. (2015)	Transcatheter aortic valve replacement	RCT	0.25 mg/kg after priming- cont.during procedure-4 h thereafter	Reduced AKI incidence rate
Katoh et al. (2019)	PCI	Prospective, observational	0.25 mg/kg, controlled during procedure up to 4 h thereafter	Prevent CI-AKI in Japanese patient with renal dysfunction

## Mannitol

Mannitol is a sugar alcohol and isomer of sorbitol so it has a sweet taste. Mannitol can be used as an osmotic diuretic and hypertonic solution which is often implemented in several clinical settings, such as crush injury, compartment syndrome, intraocular and intracranial hypertension. Mannitol has a small molecular weight that can be easily filtered at the glomerulus which then possible to reduce water reabsorption in the proximal tubule and sodium concentration. The implementation of mannitol in clinical settings has a large dose variation, as a result it can be more difficult to determine the optimal dose of mannitol use (Fandino, 2017). The mechanism of mannitol as a renal vasodilator is produced at low doses, whereas mannitol actually produces the opposite effect as a renal vasoconstrictor at high doses of administration. Acute renal failure (ARF) is very likely to occur upon administration of high doses of mannitol, where it means over than 200 g per day or cumulative dose >400 g in 48 h (Gadallah et al., 1995). Administration of mannitol in patients who have demonstrated pre-existing kidney impairment will lead to a greater potential for nephrotoxicity due to the accumulation of mannitol (Visweswaran et al., 1997). The incidence of nephrotoxicity may be caused by the use of mannitol at high serum levels (>1,000 mg/dl) in which the clinical condition can be signed by the increasing of osmolality calculated by the osmolal gap. An osmolal gap value of more than 55 mOsm/kg is very possible to increase the risk of acute kidney injury (Dorman et al., 1990; Pérez-Pérez et al., 2002; Mary et al., 2021).

## Fenoldopam

Fenoldopam is a short acting selective agonist of dopamine-1 receptors that can reduce systemic vascular resistance and improve renal blood flow which is generally used as an antihypertensive (Nichols et al., 1990). Besides the clinical benefit, fenoldopam also has a potent agonist effect on DA_1_ receptors when compared to dopamine (Murphy and ClareShorten, 2001). Both of fenoldopam and dopamine are included in dopamine receptor members that belong to G-protein-coupled receptor superfamily. The outline classification of dopamine receptor is divided in two categories, namely D_1_ and D_2_ which are located in the central nervous system and have the ability to stimulate (D_1_) or inhibit (D_2_) adenylate cyclase (Nichols et al., 1990). While the peripheral dopamine-1 receptor sites are located on blood vessels, renal tubules, and juxtaglomerular cells. At low doses, fenoldopam has a direct effect on the renal tubules which it has ability to produce natriuresis and diuresis (Ricci and Ronco, 2006) and at the renal dose, fenoldopam has no any systemic effects (Ricci and Ronco, 2006; Noce et al., 2019). In a small study, it was shown that fenoldopam exerted positive result by increasing blood flow in all renal compartments and the amelioration of renal blood flow produced in starting from a dose of 0.1–0.3 μg/kg/min with stable hemodynamic conditions of patients and preserved renal function (Meco and Cirri, 2010). Administration of fenoldopam did not provide any benefit to patient which may experience deterioration in mean arterial pressure during cardiac surgery and in the presence of insufficient of renal function. Thereby the use of fenoldopam is appropriate for the prevention strategy of postoperative AKI (Meco and Cirri, 2010; Noce et al., 2019).

## Nitric Oxide

Nitric oxide (NO) is an antioxidant and an important homeostatic mediator of renal hemodynamics. Administration of exogenous nitric oxide in CPB may be useful for maintaining organ perfusion and vasodilation due to increased release of free Hb caused by red blood cells in contact with the bypass circuit, in addition to prevent glomerular injury due to reactive oxygen species (Khorashadi et al., 2020). In an RCT study involving 244 subjects, nitric oxide administration of 80 parts per million via gas exchanger on a CPB machine followed by inhalation route for 48 h post-operative, nitric oxide can effectively reduce the incidence of AKI post-operative during the first year completion of surgery (Lei et al., 2018). In another study showed, a systematic review and meta-analysis of NO used in patients who underwent CPB is giving a promising in preventing incidence of AKI post-CPB, however it remained need further investigations to determine the time of administration and dosage regimens (Hu et al., 2019).

## N-Acetylcysteine

N-acetylcysteine (NAC) is commonly used as a mucolytic agent since its discovery several decades ago, in addition to its use as a treatment for acetaminophen toxicity. However, with the development of research in the field of pharmacology, NAC which acts as an antioxidant by reducing or even preventing oxidative stress and as a scavanger of reactive oxygen species is also used to protect kidney function and is used in the field of cardiology (Samuni et al., 2013; Pedre et al., 2021). Based on a study conducted on animals, present a form of NAC amide (NACA) which has better activity as a renoprotective agent when compared to NAC itself by upregulating thioredoxin-1 and inhibition of signal-regulating kinase 1 (ASK1) which is an activator of the p38MAPK pathway in inhibiting apoptosis of renal cells, consequently it can significantly reduce serum creatinine elevation, blood urea nitrogen, biomarkers of AKI, and prevent histologic changes resulting from renal tubular injuries (Gong et al., 2016). A pharmacokinetic study of NAC, showed that there was a significant decrease in NAC clearance of up to 90% when oral NAC was administered to patients with end-stage renal disease (ESRD), although plasma NAC levels increased dose-related (Nolin et al., 2010). The clinical use of NAC in an attempt to reduce the risk of AKI postcardiac surgery can effectively reduce the incidence of AKI based on the evidence in a systematic review and meta-analysis study, but not for all-causes of mortality and the patient’s need of RRT with high-dose intravenous NAC during the perioperative period (Tan et al., 2022; Zhao et al., 2022).

## Alteration pharmacokinetic of drugs related post-cardiopulmonary bypass utilization

In surgical procedures that use CPB, lung and heart functions are maintained by an instrument, namely the CPB machine. The use of CPB is possible to change the pharmacokinetic profile and serum concentration of drugs used during on-pump. Some of the pharmacokinetic parameters that have changed are distribution, hepatic, and renal elimination resulted by several mechanisms, namely hemodilution due to the addition of pump priming solution before initiation of CPB, coupled with low pump flow rate which causes a decrease in mean arterial pressure (MAP) resulting in changes in regional flow distribution is characterized by decline in perfusion of several organs, including several vital organs. During the use of CPB, occurrence of hypothermia may affect in decreasing of metabolic requirements (Buylaert et al., 1989).

## Future insight of technological improvement

Modification of drug delivery aims to achieve the therapeutic target as desired, which cannot be achieved by conventional methods. In administering drugs *via* intravenous injection as an infusion, modification of drug delivery can be through targeted delivery systems devices and the PK/PD approach by determining the effective plasma concentration, considering the volume of distribution, hepatic and renal elimination, and the compartmental model, in attempt to prevent accumulation of drugs which is given by infusion (Shargel et al., 2012). Enhancing device technology of drug administration, drug plasma concentrations targeted and controlled, provide feedback with high precision as the mechanism of sensors, and have capability of doses adjustment to fill the individually requirement would become a powerful tool in pharmacological research and clinical practice such as an *in-vivo* study that conducted by Netzahualcoyotl *et al* (Arroyo-Currás et al., 2018).

## Conclusion

AKI is the most common complication after cardiac surgery with CPB. The occurrence of AKI can provide a poor outcome in patients and can increase the mortality rate. Various efforts have been made to prevent the occurrence of AKI post-CPB, the focus of this article is the pharmacological strategy for the prevention of AKI, where there are four types of drugs that can work effectively by considering the patient’s physiology and the device to achieve the proper drug plasma concentration.
